# Tuberculosis tenosynovitis: A rare case report

**DOI:** 10.1590/0037-8682-0524-2020

**Published:** 2021-03-08

**Authors:** Fatma Kesmez Can, Kutsi Tuncer, Bahar Yılmaz Çankaya

**Affiliations:** 1 Ataturk University, Medical Faculty, Department of Infectious Diseases and Clinical Microbiology, Erzurum, Turkey.; 2 Ataturk University, Medical Faculty, Department of Orthopedic, Erzurum, Turkey.; 3 Ataturk University, Medical Faculty, Department of Radiology, Erzurum, Turkey.

**FIGURE 1:**
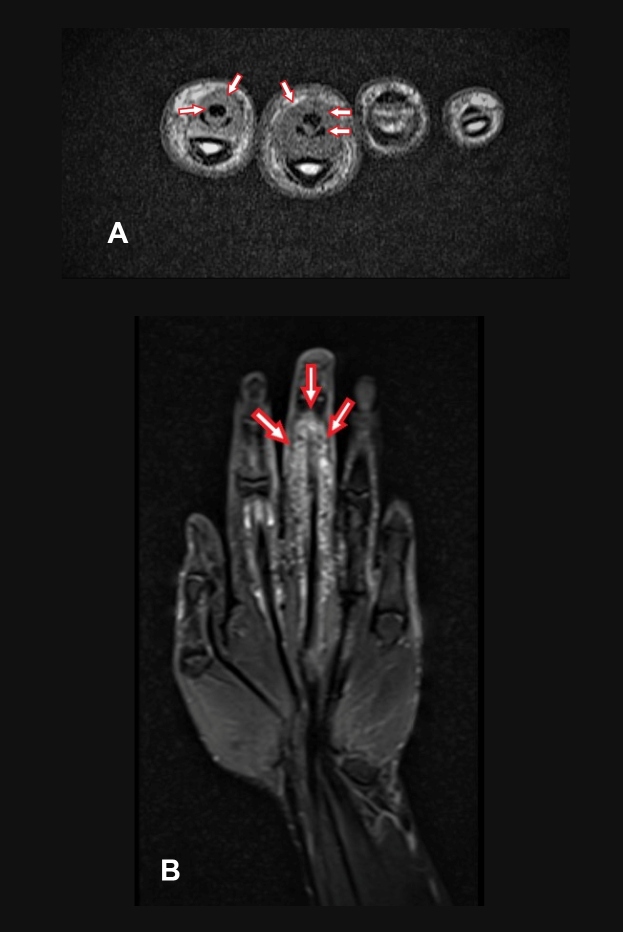
**(A)** Wrist magnetic resonance imaging. Pre-operation non-enhanced fat suppressed T2-weighted axial and coronal **(B)** images show multiple hypointense rice bodies in the enlarged synovial fluid around the digital flexor tendon sheaths of the third and index fingers (arrows).

**FIGURE 2:**
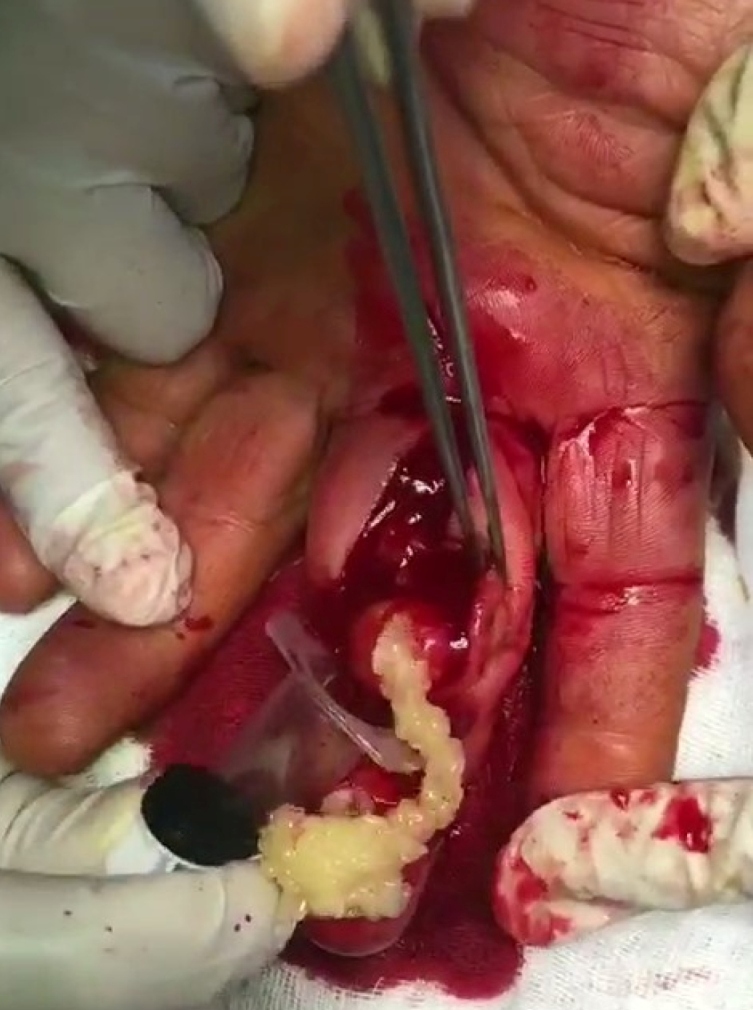
Multiple rice bodies in the third finger, which were removed during surgery

A 32-year-old man was admitted to the Infectious Diseases Outpatient Clinic of Atatürk University Medical Faculty Hospital due to swelling and pain in the third finger of his left hand that had persisted for a year. The patient worked in animal husbandry and had no other complaints. Although he had already received various drug therapies, his symptoms persisted. Brucella agglutination tests showed negative results, the purified protein derivative test showed an induration of 20 × 22 mm, and the Quantiferon test showed positive results. Magnetic resonance imaging revealed tenosynovitis in the flexor muscle tendons of the second and third fingers (Figure 1A-B). A consultation was held with the orthopedic department, after which an operation was performed on the third finger of the patient’s left hand; an excision was performed, and a sample was taken^1^ (Figure 2). 

Pathology showed necrotizing granulomatous tenosynovitis. No growth was observed in the culture for tuberculosis. Tenosynovitis due to tuberculosis was suspected. The patient was administered tuberculosis treatment consisting of isoniazid (INH), rifampicin, ethambutol, and pyrazinamide^2^ ^-^ ^3^. Quadruple therapy was applied for 2 months, and INH and rifampicin were administered for 4 months. No recurrence or residual symptoms were detected at the end of treatment. No new findings were detected at the 6-month follow-up after treatment completion.
